# Neutral boundary alignment in total knee arthroplasty: a novel concept

**DOI:** 10.1186/s40634-020-00280-4

**Published:** 2020-08-30

**Authors:** Lorenzo Deveza, Khatereh Hajizadeh, Benjamin Song, Ilwhan Park

**Affiliations:** 1 Department of Orthopaedic Surgery, Baylor College of Medicine, 7200 Cambridge Ste 10A, Houston, TX 77030 USA; 2 Lento Medical Innovation, Inc, Houston, TX USA; 3 CHA Hollywood Presbyterian Medical Center, Los Angeles, CA USA

**Keywords:** Knee alignment, Kinematic alignment, Mechanical alignment, Anatomical alignment, Functional alignment, Neutral boundary alignment, Total knee Arthroplasty

## Abstract

The goal of total knee arthroplasty (TKA) surgery is to provide a stable and functional knee joint using current implant designs. Several alignment philosophies and surgical techniques have been introduced to provide a stable and functional knee joint, such as mechanical alignment (MA), kinematic alignment (KA), and anatomical alignment (AA). Recently, functional alignment (FA) is proposed. In this concept article, we propose a TKA approach, which we termed “*Neutral Boundary Alignment (NBA)*.” The proposed approach seeks to establish the overall limb alignment in the direction of gravity at the midstance of gait cycle; consequently, a potential native knee can be restored from an arthritic state by establishing the joint line parallel to the ground. Herein, the NBA approach is described, and an iterative algorithm of structural layout patterns of truss is developed. The following three hypotheses are proposed: 1) The joint line should be parallel to the ground during the midstance of gait as an important initial condition for stability when transitioning toward gait propulsion in the gait cycle; 2) The NBA stability criteria purports that the leg is stable when the direction of gravity is simultaneously situated within the hip, knee and ankle during the midstance of gait, which generally agrees with the Varus/Valgus 3 degree envelope of MA; 3) Femoral and tibial resections that are made parallel to the ground remain within 1.5 degrees of traditional mechanical alignment resections.

## Introduction

Despite being a relatively successful surgery, patient dissatisfaction with total knee replacement has remained roughly 10–20% despite improvements in surgical approach and implant design [7, 17]. The goal of total knee arthroplasty (TKA) surgery is to provide a stable and functional knee joint. However, TKA surgery inevitably changes the natural mechanics of the knee by altering the soft tissue attachments and structures that surround the knee. Additionally, the prosthetic knee design differs in shape compared to the native knee, and this has made the optimal implant position and target leg limb alignment unclear. Indeed, coronal alignment is one of the main factors associated with TKA success and patient satisfaction. Mechanical alignment (MA) seeks a strictly biomechanically aligned knee [1, 16] as the endpoint, while kinematic alignment (KA) seeks to provide a more functionally aligned knee [12]. Anatomical alignment (AA) suggests that the optimal component position should anatomically recreate the joint line parallel to the ground [13]. Technical difficulties associated with AA led to it achieving only very limited acceptance. Recently, functional alignment (FA) is proposed as a hybrid technique to allow mechanically-sound, soft tissue-friendly alignment targets [18]. The functional alignment technique is intriguing but requires the use of computer navigation and robot assisted TKA that may hinder its widespread application [18]. However, despite significant improvements on surgical technique and in implant designs [1, 3, 4, 15], each alignment approach still has its limitations [1, 3, 4, 12, 15]; and, patient dissatisfaction still persists following TKA [7, 17].

In this concept article, we propose a total knee arthroplasty approach, which we term “*Neutral Boundary Alignment (NBA),*” which may coalesce principles of MA, KA, and AA. An iterative algorithm of structural layout patterns of truss is developed. The proposed method seeks the overall leg limb alignment in the direction of gravity and establishes the joint line parallel to the ground in ambulation, leading to restoring a potential native knee from an arthritic state.

## Methods

To help with comprehending the development of NBA, we briefly review the gait cycle (i.e. the ambulatory phase of walking). There are five phases of ambulation; 1) initial contact, 2) foot flat 3) midstance 4) heel lift and 5) toe off. The gait pattern, such as step width, step length, etc., is complex and unique to everyone. The midstance is the single limb support phase of gait cycle where the foot assumes a support and overall stability role. In an instantaneous midstance motion, the leg becomes rigid and is ready for propulsion while the center of mass is momentarily above the ankle joint. The complete sole of the foot is weight bearing as this single limb supports the entire body weight [2, 3]. In this orientation, the contact force between the femoral condyles and the tibial plateau is in compression, and the external knee adductor moment is resisted by a combination of muscle and ligament forces [21]. Therefore, the single rigid limb midstance phase provides an instant condition to apply a pseudo-static model following the minimum energy principal for the development and analysis of the NBA algorithm. Instead of a traditional MA approach, we apply structural engineering principles by the following steps: 1) identify for the hip, knee, and ankle (HKA) contact boundaries; 2) modeling of the femur and tibia as truss structures; 3) establish the neutral boundary axis (NB axis).

### Defining contact boundaries (boundary conditions)

**Fig. 1:**
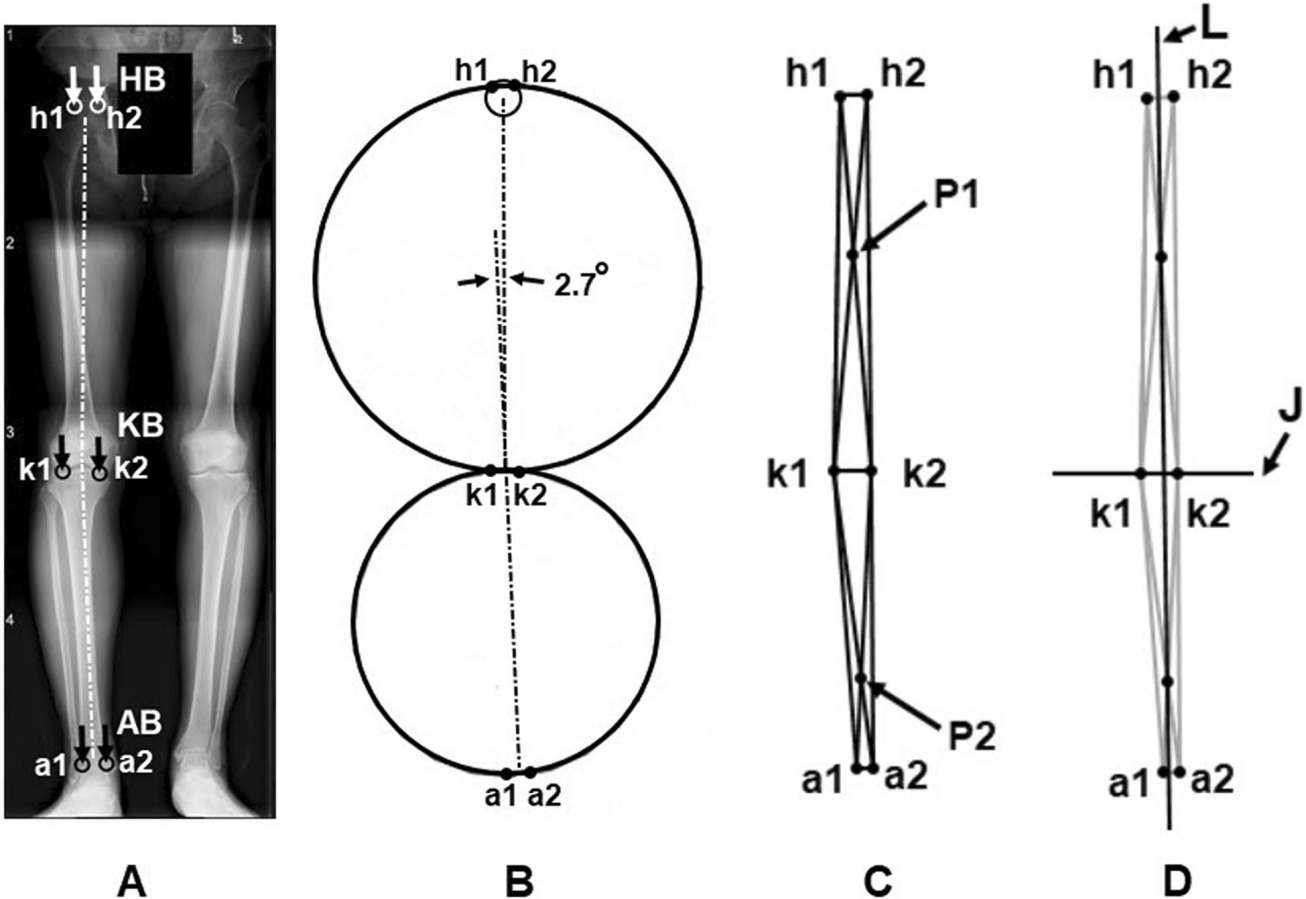
**a** Hip, knee, ankle coronal radiograph taken in midstance of gait for the right leg, which was obtained with the patient taking one-step forward and pausing for X-ray (static capture). The contact boundaries are defined in this view. **b** Based on six points representing the contact boundaries of femur and the tibia, the iterative algorithm of structural layout patterns of truss is applied. **c** A truss model is generated for the femur and tibia. **d** The NB Axis is defined by a line L connecting the intersecting points P1 and P2 and the joint line J perpendicular to NB Axis

Figure 1a shows a coronal HKA radiograph in the midstance of gait for the right leg (not a traditional two-legged standing XR). It is noted that the method could be more practically applied using a traditional two-legged standing XR, CT or MRI. The figure illustrates that the contact boundaries are defined for HKA on the radiograph in the approximate midstance phase in a coronal view. The hip contact boundary HB is defined as the contact bounds (h1, h2) of the superior surface femoral head onto the inferior surface of the acetabulum in pelvis. The knee boundary KB is defined as the contact bounds (k1, k2) in the medial and lateral femoral condyles on the tibial plateau. Finally, the ankle boundary AB is defined as the contact bounds (a1, a2) on the medial and lateral talus corners.

### Modelling of truss structure for femur and tibia

As a structure, the femur and tibia are bones with dense cortical bone and supporting internal trabecular network. It is believed that the trabecular bone is aligned within the stress lines of the femur, especially, at the femoral calcar [22, 23]. It is recognized that the femur and tibia are more than shells of cortical bone, as the trabecula provide an internal truss for the bone, mechanically providing strength at the lowest weight possible. For this reason, we infer the femur and tibia can be modeled as a truss structure [20].

An iterative algorithm of structural layout patterns of truss is developed. This approach includes Ptolemy’s theorem [10] and Delaunay and Voronoi tessellations [9, 11]. Ptolemy’s theorem is a relation between the four sides and two diagonals of a cyclic quadrilateral. Ptolemy’s theorem is applied to constrain the HKA boundary points. Further, the structural layout patterns are a truss as Delaunay and Voronoi tessellations and are applied for the truss connectivity of the HKA boundaries (Fig. 1b). Figure 1c exhibits the construction of femoral and tibial quadrilaterals as trusses with diagonals. The intersecting points of the femoral and tibial diagonals are defined as *P1* and *P2*, where the points *P1* and *P2* represent the center points of femoral and tibial quadrilaterals, respectively.

### Establishing neutral boundary Axis

Once the femoral and tibial trusses are constructed, the straight-line *L*, connecting the points of *P1* to *P2*, is defined as “Neutral Boundary Axis” in Fig. 1d. Although an individual’s gait cycle is unique, he or she prefers to move in an optimal way of walking. This implies that the NB axis should follow the direction of gravity according to the minimum energy principal [2] in the midstance phase. Consequently, aligning the NB axis in the direction of gravity is an important factor in determining the overall leg limb alignment of the native knee, while the NB axis being situated within the HKA boundaries for the single limb support is important to prevent Varus/Valgus (V/V) instability. These conditions assure structural stability and knee balance in the midstance phase during ambulation.

### Defining joint line (initial condition)

Figure 1d exhibits the joint line *J* (the femoral axis of initial knee flexing) and is defined as the line perpendicular to the NB axis (which is in the direction gravity). This leads to inevitable conclusion that the joint line is parallel to the ground [13, 14], which is assumed to be one shared feature for everyone. Despite the complex dynamic knee motion, the joint line being parallel to the ground is of significance as the initial condition in the knee structure when ready for a transition toward propulsion in gait cycle.

## Discussion/conclusion

**Fig. 2:**
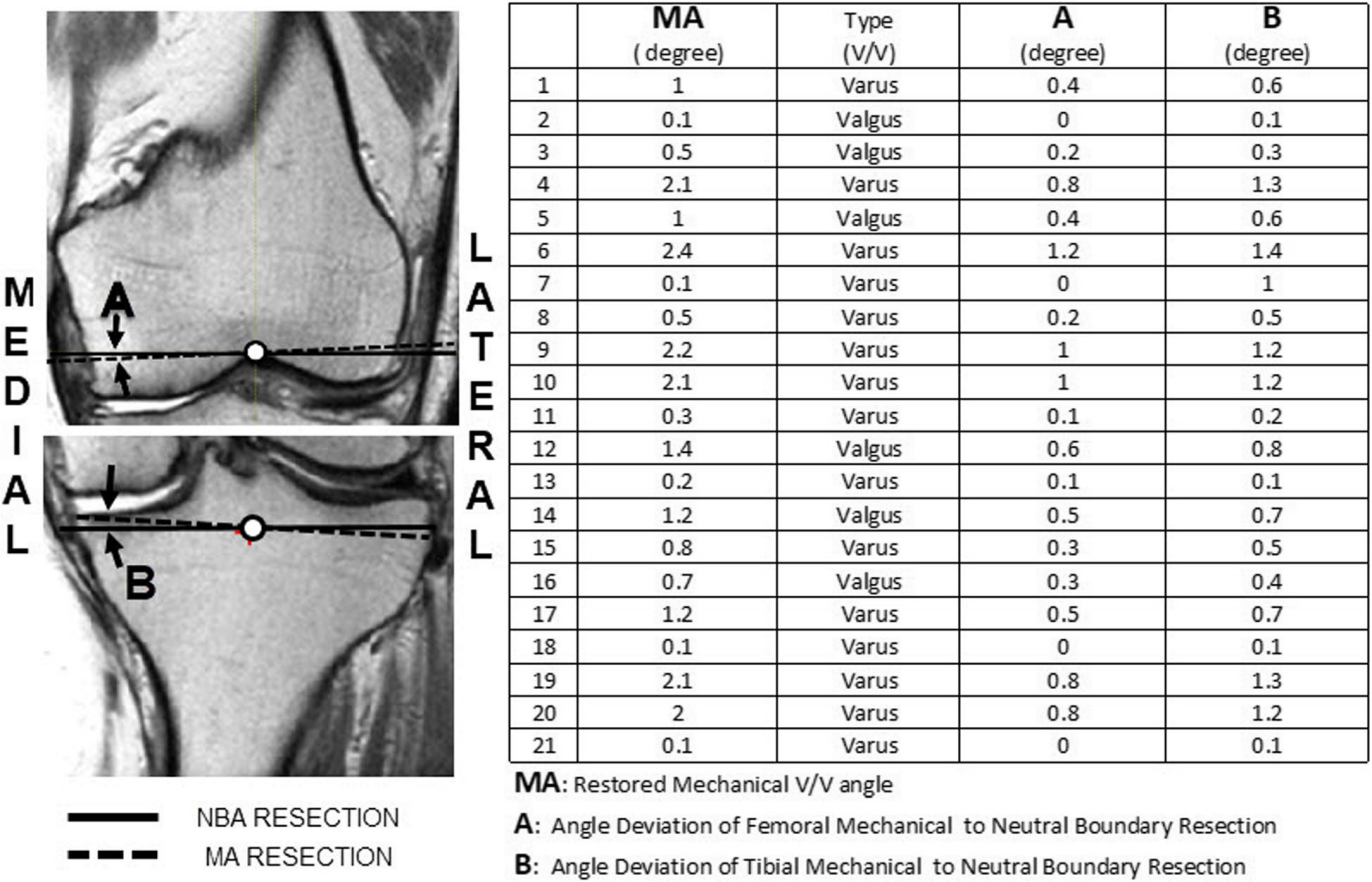
Restored varus/valgus angles of 21 arthritis patients using MRI scans of the hip, knee and ankle obtained in a single global coordinate system and in the supine position. MA is determined by finding the femoral MA and tibial MA separately. Boundary conditions for the NBA approach are defined for the hip, knee and ankle and the NB axis is defined as described in the article. Angle Deviations of Femoral and Tibial MA to NBA Resections are reported. The NBA combined femoral and tibial cuts are oriented parallel to the ground and maintain global mechanical alignment stability

In this concept article, NBA modeling is proposed and the following hypotheses are made:

1. The joint line being parallel to the ground (femoral axis of initial knee flexing) is significant as an initial condition for the midstance in ambulation ready for a transition toward propulsion. Hence, this should be included as one of the TKA requirements that assures the overall leg limb alignment in the direction of gravity [13].
2. Varus/Valgus (V/V) 3 degree envelope has been widely accepted as the biomechanical stability condition of TKA [5, 6, 8, 16, 19]. Herein, we define the knee stability and balance condition as “if the neutral boundary axis is in the direction of gravity and is simultaneously situated within hip, knee, and ankle boundaries in the midstance phase of the gait cycle, then the knee is set to be stable and balanced.” Fig. 2 illustrates that the V/V angles of the restored native leg limb alignment fall within V/V 3 degrees using the proposed iterative algorithm for 21 patients as assessed by HKA MRI images. Due to the narrow dimension of the ankle boundary as the base of a single limb support, the NBA stability condition may generally agree with the V/V 3-degree envelope. However, the NBA stability criteria is not an absolute condition for everyone, but rather a relative one depending on individual’s anatomical structure (i.e., boundary conditions).
3. It is another observation from Fig. 2 that NBA resections of the femur and tibia could be obtained with the angle deviation range of 0 to 1.5 degree with respect to MA resection angles in conjunction with patient’s V/V angle information, which may support the concept of functional alignment or similar techniques [3, 18].

It is important to note that the results and hypothesis of this concept article may not be applicable to those who have walking abnormalities prior to osteoarthritis. For example, it is contraindicated in those with femoral or tibial deformities, leg length discrepancies or those with neuromuscular disorders, but this does not include those that did not have a gait disturbance prior to the development of osteoarthritis. The NBA approach would be a useful pre-operative planning tool, providing surgeons with valuable surgical information prior to TKA in order to attain the knee alignment based on the individual’s knee characteristics prior to osteoarthritis, and hence, we speculate that this might lead to higher rate of patient satisfaction. As this article presents a hypothesis, long-term studies, including a clinical study are required to confirm the NBA approach. Also, future studies should include extending the NBA algorithm to modeling in three-dimensions by including sagittal direction. This can provide the understanding of a tibial slope in TKA.

## Data Availability

Not applicable.

## Abbreviations

NBA: Neutral Boundary Alignment
MA: Mechanical Alignment
KA: Kinematic Alignment
FA: Functional Alignment
HKA: Hip-Knee-Ankle
TKA: Total Knee Arthroplasty
V/V: Varus/Valgus
NB: Neutral Boundary
